# High prevalence of asymptomatic malaria in south-eastern Bangladesh

**DOI:** 10.1186/1475-2875-13-16

**Published:** 2014-01-09

**Authors:** Peter Starzengruber, Hans-Peter Fuehrer, Benedikt Ley, Kamala Thriemer, Paul Swoboda, Verena Elisabeth Habler, Mariella Jung, Wolfgang Graninger, Wasif A Khan, Rashidul Haque, Harald Noedl

**Affiliations:** 1 Institute of Specific Prophylaxis and Tropical Medicine, Medical University of Vienna, Kinderspitalgasse 15, Vienna 1090, Austria; 2 MARIB, Malaria Research Initiative Bandarban, Bandarban, Bangladesh; 3 Department of Internal Medicine I, Division of Infectious Diseases and Tropical Medicine, Medical University of Vienna, Vienna, Austria; 4 Department of Pathobiology, Institute of Parasitology, University of Veterinary Medicine Vienna, Vienna, Austria; 5 International Centre for Diarrhoeal Disease Research Bangladesh, Dhaka, Bangladesh

**Keywords:** Malaria, Prevalence, Asymptomatic, Bangladesh, PCR, Microscopy

## Abstract

**Background:**

The WHO has reported that RDT and microscopy-confirmed malaria cases have declined in recent years. However, it is still unclear if this reflects a real decrease in incidence in Bangladesh, as particularly the hilly and forested areas of the Chittagong Hill Tract (CHT) Districts report more than 80% of all cases and deaths. surveillance and epidemiological data on malaria from the CHT are limited; existing data report *Plasmodium falciparum* and *Plasmodium vivax* as the dominant species.

**Methods:**

A cross-sectional survey was conducted in the District of Bandarban, the southernmost of the three Hill Tracts Districts, to collect district-wide malaria prevalence data from one of the regions with the highest malaria endemicity in Bangladesh. A multistage cluster sampling technique was used to collect blood samples from febrile and afebrile participants and malaria microscopy and standardized nested PCR for diagnosis were performed. Demographic data, vital signs and splenomegaly were recorded.

**Results:**

Malaria prevalence across all subdistricts in the monsoon season was 30.7% (95% CI: 28.3-33.2) and 14.2% (95% CI: 12.5-16.2) by PCR and microscopy, respectively. *Plasmodium falciparum* mono-infections accounted for 58.9%, *P. vivax* mono-infections for 13.6%, *Plasmodium malariae* for 1.8%, and *Plasmodium ovale* for 1.4% of all positive cases. In 24.4% of all cases mixed infections were identified by PCR. The proportion of asymptomatic infections among PCR-confirmed cases was 77.0%, oligosymptomatic and symptomatic cases accounted for only 19.8 and 3.2%, respectively. Significantly (p < 0.01) more asymptomatic cases were recorded among participants older than 15 years as compared to younger participants, whereas prevalence and parasite density were significantly (p < 0.01) higher in patients younger than 15 years. Spleen rate and malaria prevalence in two to nine year olds were 18.6 and 34.6%, respectively. No significant difference in malaria prevalence and parasite density was observed between dry and rainy season.

**Conclusions:**

A large proportion of asymptomatic plasmodial infections was found which likely act as a reservoir of transmission. This has major implications for ongoing malaria control programmes that are based on the treatment of symptomatic patients. These findings highlight the need for new intervention strategies targeting asymptomatic carriers.

## Background

The World Health Organization (WHO) estimated 660,000 deaths in 2011 directly attributed to malaria, approximately half of the world’s population being at risk of infection [1]. The disease has re-emerged in several Central Asian countries and in Southeast Asia partly because of relenting malaria control efforts and the emergence of parasite resistance to the most commonly used anti-malarial drugs [2]. In many regions the vectors have become resistant to the main insecticides and cases of artemisinin resistance have been reported from the Greater Mekong subregion [3-6].

Resistance to chloroquine and sulphadoxine/pyrimethamine (S/P) has been reported from Bangladesh [1] but until now there is no evidence that artemisinin resistance has spread westwards to Bangladesh, which traditionally forms a gateway to the Indian Subcontinent [7].

In 2004 the Ministry of Public Health and Family Welfare of Bangladesh revised the malaria treatment guidelines, introducing artemisinin-based combination therapy (ACT) in areas with resistance against chloroquine and S/P [1]. However, ACT was not deployed on a major scale until 2007. Despite the introduction and distribution of ACT in the last years the WHO reports a 70% increase in case numbers between 2000 and 2010 in Bangladesh. However, it is difficult to discern the underlying trend in malaria incidence from improved reporting due to continuous improvements in diagnostic facilities [8]. A seemingly contradictory statement was given in the 2012 report where a decrease of 69% in malaria case incidence between 2000 and 2011 has been reported [1]. Due to a shortage of staff in health care facilities and shortcomings in surveillance and information systems there is still a significant lack of data on malaria from this area [9,10]. Further increasing drug resistance is significantly aggravating the malaria situation and treatment alternatives to currently used anti-malarials are missing [11-14].

In 2007 a rapid diagnostic test (RDT)-based, cross-sectional survey in 13 eastern districts of the country showed malaria to be endemic within the entire study area. Overall prevalence was reported to be approximately 4%, the majority of cases (90.2%) due to *Plasmodium falciparum. Plasmodium vivax* and mixed infections accounted for only 5.3%and 4.5%, respectively. Highest prevalence rates were reported from the Chittagong Hill Tract (CHT) with up to 15% [15]. Surveys among febrile patients in the region showed a malaria positivity rate of 26% by microscopy (Swoboda *et al.* personal communication), which increases to 50% when using polymerase chain reaction (PCR), a considerably more sensitive diagnostic tool [16].

The high sensitivity of PCR allows detecting subpatent infections that are frequently asymptomatic and it has been shown that those undetected infections represent a considerable fraction of overall infections and may therefore act as a reservoir for transmission [17-21]. The aim of this study was therefore to assess prevalence and proportion of asymptomatic *P. falciparum* infections in the southernmost district of the CHTs.

## Methods

### Study setting and procedure

Two cross-sectional surveys were performed: the first during the rainy season from August to October 2007 and the second during the dry season from December 2007 to February 2008. The study was conducted by a team from the Medical University of Vienna, Austria in collaboration with the International Centre for Diarrhoeal Disease Research, Dhaka, Bangladesh (ICDDR, B). All selected communities were visited by members of the study team, prior to sample collection to inform villagers about the ongoing study. Laboratory tests on collected samples and data analysis were carried out at the Malaria Research Initiative Bandarban (MARIB) field research centre in Bandarban town. Written informed consent was obtained from all study participants or their legal representative, the study protocol was approved by the Ethical Review Committee of the International Centre for Diarrhoeal Disease Research, Bangladesh (ICDDR, B).

### Sampling

**Figure 1:**
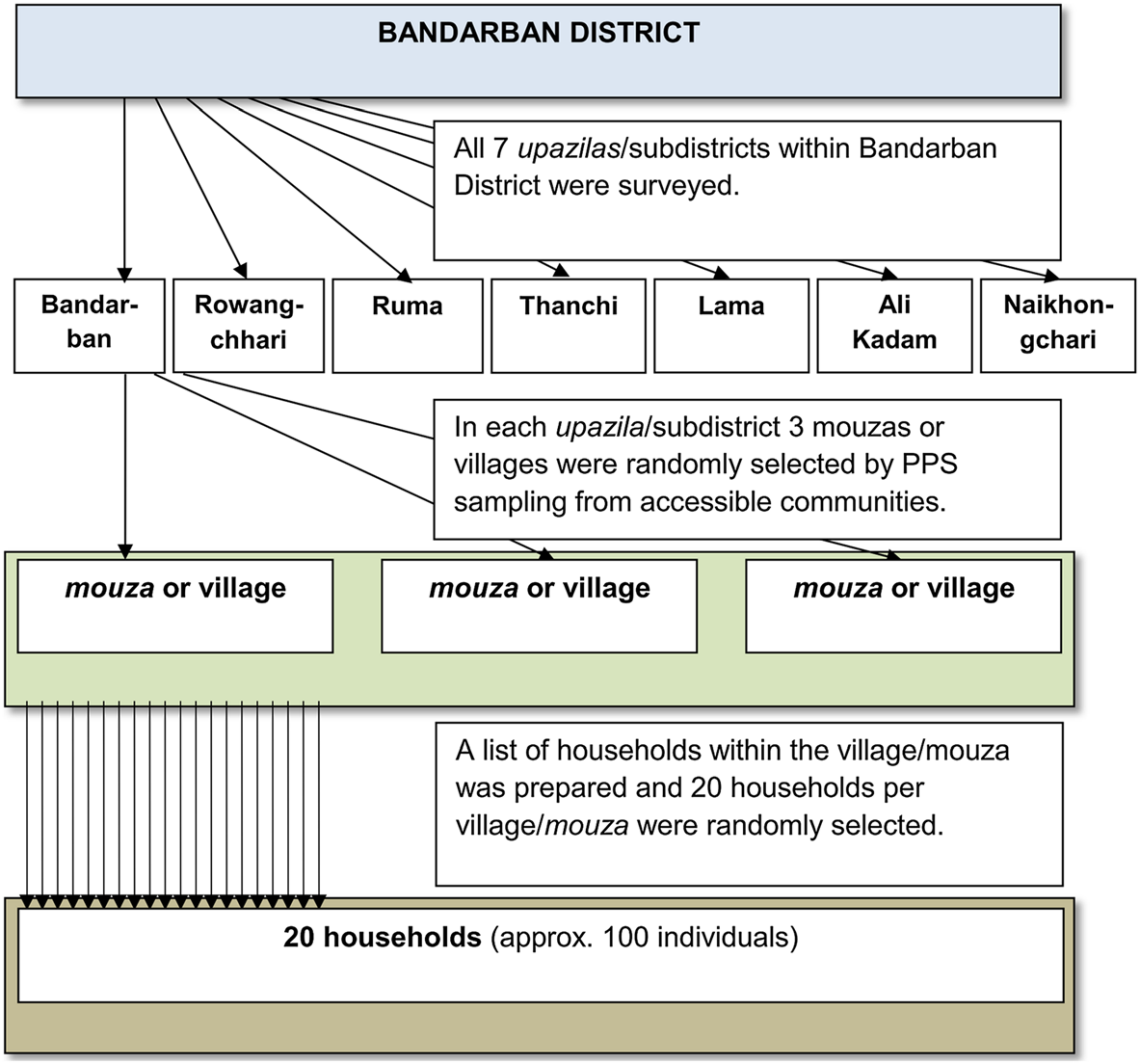
**Flow chart for selecting clusters and households within Bandarban District.** *PPS (probability proportional to size).

Administratively, Bandarban District consists of seven subdistricts (*upazilas*), 32 unions, 140 mouzas and 1,482 villages. Geographical multistage cluster sampling techniques in a single domain were employed (Figure 1) using population figures from the 2001 census [22]. For each of the seven subdistricts all mouzas or villages were listed alphabetically and 3 mouzas or villages were randomly selected using a probability proportion to size (PPS) sampling procedure. A list of all households within the mouza or village was prepared and 20 households per mouza or village were randomly selected. All the persons present in the household were invited to participate in the survey.

During the monsoon season 2007 a total of 21 villages in all seven subdistricts were surveyed (Survey I). Eight villages in three subdistricts (Bandarban, Ruma and Rowangchari) were revisited during the second survey period (Survey II). Whenever possible the study team visited the same households as in the previous survey. Household members who declined to participate or had moved away from the area were replaced by their closest neighbouring household.

### Data and sample collection

Participants were interviewed and information was recorded on gender, age, ethnicity, occupation, height, weight, history of fever, number of previous episodes of malaria/fever, last date of malaria diagnosis and result of diagnosis, source and drugs used in malaria treatment, and the number of household members. Axillary temperature and pulse were measured in all participants. Three ml of venous blood was collected from participants eight years or older for malaria RDT/microscopy and 100 μl of venous blood transferred on filter paper (903; Schleicher & Schuell, BioScience GmbH, Dassel, Germany) in duplicate. Five drops of blood (one drop of blood for the malaria RDTs, two for microscopy, and two drops of blood for the PCR filter paper) were drawn by finger or heel prick from children below eight years old.

### Laboratory methods

Malaria was diagnosed in the field by RDT and later confirmed by microscopy and PCR.

### Rapid diagnostic test

RDTs (FalciVax^®^, Zephyr Biomedicals, India) based on the detection of *P. falciparum*-specific histidine-rich protein 2 (HRP2) and *P. vivax*-specific lactate dehydrogenase (Pv-pLDH) were employed in all participants with malaria-like symptoms [23,24]. Patients who tested positive were provided with immediate treatment following national guidelines. Febrile patients considered seriously ill were immediately referred to the closest health care facility for further diagnosis and treatment.

### Microscopy

Giemsa-stained blood smears were used for microscopic diagnosis of malaria following established standard operating procedures (SOPs). Thick and thin blood films were prepared, stained with Giemsa stain (Merck^®^, Darmstadt, Germany) and examined under an oil immersion (Olympus microscope CX21, Tokyo, Japan) for parasite positivity and species determination. Declaring a slide positive or negative and initial species diagnosis was based on the examination of 200 fields in thick films. A slide was considered positive when at least one parasite was found. After finding the first parasite another 200 fields were completed to rule out mixed infections. If no parasite was found in 200 oil fields the slide was considered negative. Parasite density was calculated by counting the number of asexual malaria parasites per 200 white blood cells (WBCs) assuming a WBC count of 8,000/μL [25].

### Quality control

A minimum of 5% of all positive and negative slides were randomly selected for internal quality control.

### Polymerase chain reaction (PCR)

PCR was performed to assess the malaria prevalence in both, symptomatic and asymptomatic participants. DNA was isolated from filter papers using a modified chelex-based technique [26,27]. Nested PCR assays and *Plasmodium* genus-specific primers rPLU1/rPLU5 were used for the Nest1 amplification and rPLU3/rPLU4 for Nest2 reactions as described previously [28-30]. All genus-specific PCR-positive results were analysed to species level using the following species-specific primers as reported previously: rFAL1/rFAL2 (*P. falciparum*); rVIV1/rVIV2 (*P. vivax*); rMAL1/rMAL2 (*P. malariae*); rOVA1WC/rOVA2WC (*Plasmodium ovale curtisi* and *Plasmodium ovale wallikeri*); rOVA1/rOVA2 (*P. ovale curtisi*); rOVA1var/rOVA2var (*P. ovale wallikeri*) [28-33].

### Case definition

Symptomatic clinical malaria cases were defined as PCR-positive individuals with documented fever (axillary temperature ≥37.5°C) and reported clinical symptoms consistent with malaria in the previous seven days. Oligo-symptomatic malaria cases were defined as PCR-positive, afebrile (axillary temperature <37.5°C) cases with a reported history of fever or illness in the previous seven days. Asymptomatic malaria cases were defined as PCR-positive cases without measurable fever (axillary temperature <37.5°C), who reported no malaria-related symptoms, and had not received treatment for malaria in the previous seven days [34].

### Splenomegaly

The spleen rate was determined for children aged 14 years and below. Spleen size was measured followingthe method established by Hackett [35] and classified either as negative (Hackett grade 0) or positive (Hackett grades 1–5).

### Classification of malaria endemicity

The four classes of endemic malaria were defined as following [35]: hypo-endemic malaria: spleen and/or parasite rate in children two to nine years not exceeding 10%; meso-endemic malaria: spleen and/or parasite rate rate in children two to nine years between 11 and 50%; hyperendemic malaria: spleen and/or parasite rate rate in children two to nine years constantly over 50% and a high spleen and/or parasite rate rate in adults (over 25%)*;* holo-endemic malaria: spleen and/or parasite rate rate in children two to nine years constantly over 75%, but a low spleen and/or parasite rate rate in adults.

### Statistical analysis

P < 0.05 was considered significant. Pearson’s Chi-square with Yates’ correction or Fisher’s Exact test as appropriate were used for categorical data, Mann–Whitney *U*-test was performed for comparing continuous data that did not conform to normal distribution. Student’s *t*-test was conducted to evaluate differences between quantitative variables that were normally distributed. All analyses were performed using Microsoft Excel^®^ and VassarStats [36]*.*

## Results

### Demographic data of the study population

A total of 1,418 individuals in 416 households from 21 villages were included in the first survey and 436 were revisited and included in the second survey. The male/female ratio was 0.84. The median age was 24 years (IQR 9–40). The distribution of the different age groups is shown in Table 1.

**Table 1:** Baseline characteristics of the study population

**Total number of individuals 1,874**		
	**Summer survey I**	**Winter survey II**
No of individuals	1,418	436
Sex		
Male	647	178
Female	771	258
Median age (IQR)	24 (9–40)	29 (12–45)
Age groups (years)	Male/female	Male/female
0-7	133/137 (19.0%)	42/45 (20.0%)
8-14	115/133 (17.5%)	20/19 (8.9%)
15-21	57/98 (10.9%)	10/28 (8.7%)
22-50	254/318 (40.3%)	72/127 (45.6%)
>50	88/85 (12.2%)	34/39 (16.7%)
Mean weight kg (range)	39 (5–96)	41 (7–113)
Mean height cm (range)	138 (50–180)	140 (50–190)
Occupations		
None (students, children)	551 (38.9%)	
Farmer	546 (38.5%)	
Household	165 (11.6%)	
Business/service	57 (4.0%)	
Unemployed/retired	38 (2.7%)	
Others	61 (4.3%)	

### Malaria prevalence and fever in cross-sectional survey I

**District-wide malaria prevalence** (Table 2)*:* Malaria prevalence across all subdistricts in the monsoon season was 30.7% (95% CI: 28.3-33.2) and 14.2% (95% CI: 12.5-16.2) by PCR and microscopy, respectively. Of the 1,418 samples collected 435 (30.7%, 95% CI: 28.3-33.2) were PCR-positive. *Plasmodium falciparum* accounted for 58.9% (N = 256, 95% CI: 54.1-63.5), *P. vivax* for 13.6% (N = 59, 95% CI: 10.6-17.2), *P. malariae* for 1.8% (N = 8, 95% CI: 0.9-3.7) and *P. ovale* for 1.4% (N = 6, 95% CI: 0.6-3.1) of all malaria-positive individuals by PCR. The remaining 24.4% (N = 106, 95% CI: 20.5-28.7) were mixed infections. *Plasmodium falciparum* gametocytes were detected by microscopy in 11 (2.5%, 95% CI: 1.3-4.6) individuals, seven of whom had only gametocytes.

**Table 2:** Malaria prevalence, parasite density in Bandarban District (Survey I)

**Bandarban district**							
		**PCR/Microscopy**					
	**No.**	***P.f.***	***P.v.***	***P.m.***	***P.o.***	**Mixed**	**Negative**
**Sex**							
Male	647	122/77 (18.9/11.9%)	26/17 (4.0/2.6%)	3/0 (0.5/0.0%)	3/0 (0.5/0.0%)	66/3 (10.2/0.5%)	427/550 (66.0/85.0%)
Female	771	134/84 (17.4/10.9%)	33/20 (4.3/2.6%)	5/0 (0.6/0.0%)	3/0 (0.4/0.0%)	40/1 (5.2/0.1%)	556/666 (72.1/86.4%)
**Male/Age** (years)							
0-7	133	17/20 (12.8/15.0%)	9/6 (6.8/4.5%)	0/0 (0.0/0.0%)	0/0 (0.0/0.0%)	7/1 (5.3/0.8%)	100/106 (75.2/79.7%)
8-14	115	25/25 (21.7/21.7%)	5/2 (4.3/1.7%)	1/0 (0.9/0.0%)	0/0 (0.0/0.0%)	23/1 (20.0/0.9%)	61/87 (53.0/75.7%)
15-21	57	15/6 (26.3/10.5%)	0/4 (0.0/7.0%)	0/0 (0.0/0.0%)	0/0 (0.0/0.0%)	5/0 (8.8/0.0%)	37/47 (64.9/82.5%)
22-50	254	49/20 (19.3/7.9%)	11/4 (4.3/1.6%)	1/0 (0.4/0.0%)	3/0 (1.2/0.0%)	27/1 (10.6/0.4%)	163/229 (64.2/90.2%)
>50	88	16/6 (18.2/6.8%)	1/1 (1.1/1.1%)	1/0 (1.1/0.0%)	0/0 (0.0/0.0%)	4/0 (4.5/0.0%)	66/81 (75/92.0%)
**Female/Age** (years)							
0-7	137	26/30 (19.0/21.9%)	10/8 (7.3/5.8%)	1/0 (0.7/0.0%)	1/0 (0.7/0.0%)	12/0 (8.8/0.0%)	87/99 (63.5/72.3%)
8-14	133	30/25 (22.6/18.8%)	6/5 (4.5/3.8%)	1/0 (0.8/0.0%)	0/0 (0.0/0.0%)	14/1 (10.5/0.8%)	82/102 (61.7/76.7%)
15-21	98	23/5 (23.5/5.1%)	2/2 (2.0/2.0%)	1/0 (1.0/0.0%)	0/0 (0.0/0.0%)	4/0 (4.1/0%)	68/91 (69.4/92.9%)
22-50	318	47/22 (14.8/6.9%)	13/5 (4.1/1.6%)	2/0 (0.6/0.0%)	1/0 (0.3/0.0%)	10/0 (3.1/0.0%)	245/291 (77.0/91.5%)
>50	85	8/2 (9.4/2.4%)	2/0 (2.4/0.0%)	0/0 (0.0/0.0%)	1/0 (1.2/0.0%)	0/0 (0.0/0.0%)	74/83 (87.1/97.6%)
**Parasite density/Age** (years)		0–7	8–14		15–21	22–50	>50
*P. f.* (parasites/μl blood^1^)		993	954		388	525	220
*P. v.* (parasites/μl blood^1^)		425	407		263	346	960^2^
**Subdistrict**							
Rowangchhari	184	50/31 (27.2/16.8%)	10/9 (5.4/4.9%)	0/0 (0.0/0.0%)	1/0 (0.5/0.0%)	29/1 (15.8/0.5%)	94/143 (51.1/77.7%)
Thanchi	218	62/41 (28.4/18.8%)	15/7 (6.9/3.2%)	0/0 (0.0/0.0%)	1/0 (0.5/0.0%)	21/0 (9.6/0.0%)	119/170 (54.6/78.0%)
Bandarban Sadar	219	52/27 (23.7/12.3%)	6/7 (2.7/3.2%)	2/0 (0.9/0.0%)	0/0 (0.0/0.0%)	18/3 (8.2/1.4%)	141/182 (64.4/83.1%)
Ruma	212	41/19 (19.3/9.0%)	11/5 (5.2/2.4%)	4/0 (1.9/0.0%)	1/0 (0.5/0.0%)	15/0 (7.1/0.0%)	140/188 (66.0/88.7%)
Naikhongchhari	181	18/16 (9.9/8.8%)	9/4 (5.0/2.2%)	1/0 (0.6/0.0%)	3/0 (1.7/0.0%)	9/0 (5.0/0.0%)	141/161 (77.9/89.0%)
Lama	215	21/17 (9.8/7.9%)	4/3 (1.9/1.4%)	1/0 (0.5/0.0%)	0/0 (0.0/0.0%)	8/0 (3.7/0.0%)	181/195 (84.2/90.7%)
Ali Kadam	189	12/10 (6.3/5.3%)	4/2 (1.9/0.9%)	0/0 (0.0/0.0%)	0/0 (0.0/0.0%)	6/0 (2.8/0.0%)	167/177 (88.4/93.7%)

^1^Geometric mean.

^2^n = 1.

With 188 cases (36.3%, 95% CI: 32.2-40.6) the malaria prevalence in individuals under 15 years was significant higher (p < 0.01) compared to older participants (N = 247, 27.4%, 95% CI: 24.6-30.5). A significantly higher malaria prevalence (p = 0.015) was found in males (N = 220, 34.0%, 95% CI: 30.4-37.8) as compared to females (N = 215, 27.9%, 95% CI: 24.8-31.2).

The overall geometric mean parasite density determined by microscopy was 707 parasites/μl (95% CI: 565–883) for *P. falciparum* and 345 parasites/μl (95% CI: 246–484) for *P. vivax*. Parasite densities in individuals younger than 15 years were significantly higher (p < 0.01).

Out of a total of 435 PCR malaria-positive individuals 335 (77.0%, 95% CI: 72.7-80.8) were classified as asymptomatic, 86 (19.8%, 95% CI: 16.2-23.9) as oligosymptomatic, and 14 (3.2%, 95% CI: 1.8-5.5) symptomatic cases were found. Significantly (p < 0.01) more asymptomatic cases (N = 208) were recorded among participants older than 15 years compared to younger individuals (n = 127). In individuals infected with *P. falciparum* or *P. vivax* no difference (P > 0.05) was found between the proportion of asymptomatic and oligosymptomatic clinical cases (Table 3).

**Table 3:** Distribution of symptomatic, oligosymptomatic and asymptomatic PCR/microscopy positive malaria cases during monsoon and dry season

**Survey I (monsoon season)**	**Symptomatic malaria cases**	**Oligosymtomatic malaria cases**	**Asymptomatic malaria cases**
*P. f.*	11/5 (4.3/3.1%)	52/41 (20.3/25.5%)	193/115 (75.4/71.4%)
*P. v.*	2/1 (3.4/2.7%)	12/8 (20.3/21.6%)	45/28 (76.3/75.7%)
*P. m.*	0/0 (0.0/0.0%)	1/0 (12.5/0.0%)	7/0 (87.5/0.0%)
*P. o.*	0/0 (0.0/0.0%)	2/0 (33.3/0.0%)	4/0 (66.7/0.0%)
Mixed infections	1/0 (0.0/0.9%)	19/1 (17.9/25.0%)	86/3 (81.1/75.0%)
Total	14/6 (3.2/3.0%)	86/50 (19.8/24.7%)	335/146 (77.0/72.3%)
**Survey II (dry season)**			
*P. f.*	0/0 (0.0/0.0%)	10/2 (14.7/8.0%)	58/23 (85.3/92.0%)
*P. v.*	0/0 (0.0/0.0%)	3/2 (12.0/28.6%)	22/5 (88.0/71.4%)
*P. m.*	0/0 (0.0/0.0%)	0/0 (0.0/0.0%)	5/0 (0.0/100%)
*P. o.*	0/0 (0.0/0.0%)	0/0 (0.0/0.0%)	1/0 (0.0/100%)
Mixed infections	0/0 (0.0/0.0%)	0/0 (0.0/0.0%)	17/3 (100/100%)
Total	0/0 (0.0/0.0%)	13/4 (11.2/11.4%)	103/31 (88.8/88.6%)

Out of 416 households in 223 (53.6%, 95% CI: 48.7-58.5) at least one member of the household tested positive for malaria, in 64 households (15.4%, 95% CI: 12.1-19.3) two people tested positive for malaria and in 56 (13.5%, 95% CI: 10.4-17.2) households three or more individuals tested positive by PCR.

### Subdistricts/*Upazilas*

**Figure 2:**
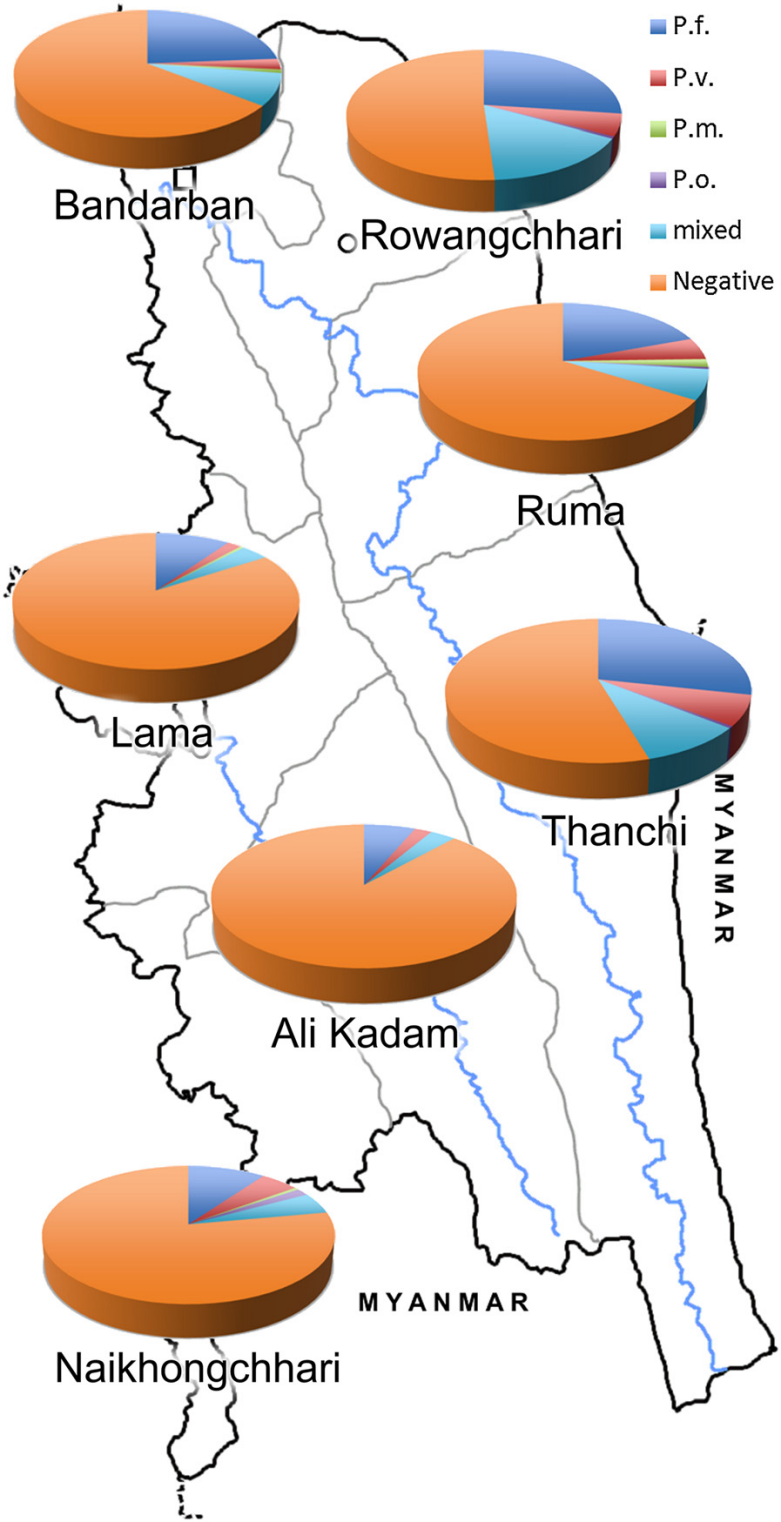
Overall malaria prevalence in Bandarban District, southeast Bangladesh during the rainy season 2007.

*Plasmodium falciparum* was the predominant species in all subdistricts*.* The highest malaria prevalence was found in the eastern subdistricts Rowangchari and Thanchi, followed by Bandarban Sadar, Ruma, Naikhongchhari, and Lama. The lowest malaria prevalence was detected in Ali Kadam, which shares only a short stretch of border with Myanmar. Malaria prevalence among the seven subdistricts is shown in detail in Table 2. Significantly higher malaria prevalence (p < 0.01) was seen in the northern and northeastern subdistricts (Bandarban Sadar, Rowangchhari, Ruma, and Thanchi) located in the foothills close to the Indian/Myanmar boarder compared with the western subdistricts (Lama, Ali Kadam and Naikhongchhari) in or closer to the plains (Figure 2). At village level the highest malaria prevalence by PCR with 71.4% (95% CI: 53.5-84.8) was found in Bagan village in Ruma subdistrict and the lowest with 1.2% (95% CI: 0.06-7.3) in Memberpara village in Lama subdistrict (p < 0.0001).

### Spleen rate

**Figure 3:**
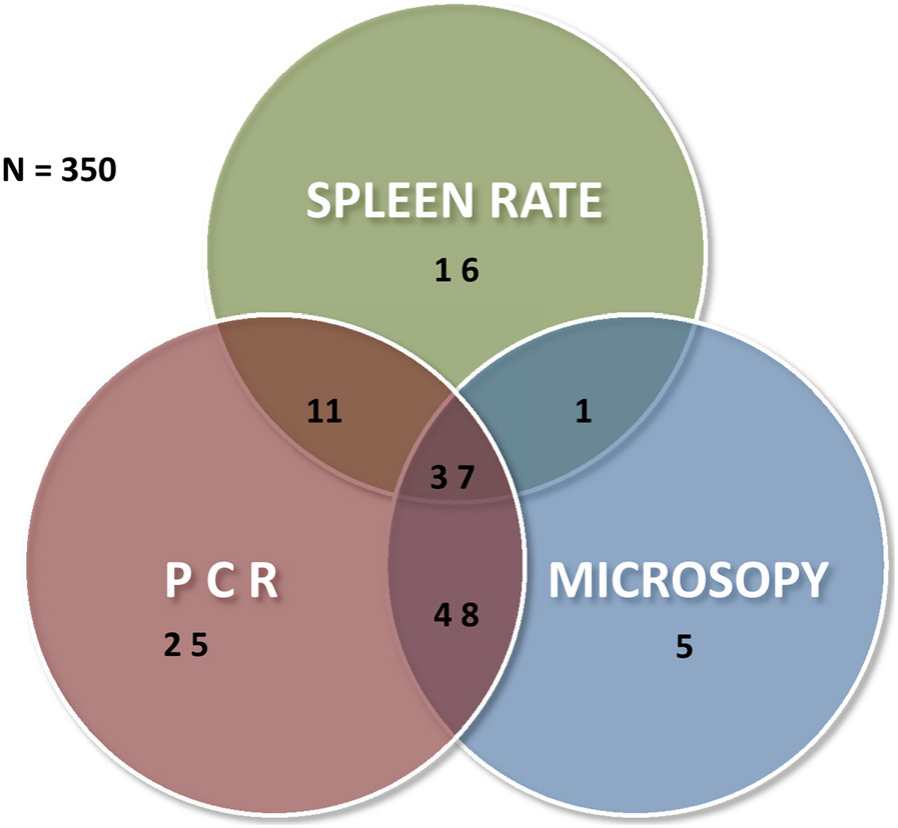
Venn diagram showing relationship between spleen rate, microscopy and PCR positive children aged two to nine years.

Out of a total of 350 children aged two to nine years, 65 had a palpable spleen (spleen rate: 18.6%, 95% CI: 14.7-23.1); 91 of them (26.0%, 95% CI: 21.6-31.0) were slide-positive for malaria either for *P. falciparum* or *P. vivax.* A total of 121 (34.6%, 95% CI: 29.6-39.8) children tested positive by PCR for any of the four malaria species. Thirty-eight (10.9%, 95% CI: 7.9-14.7) and 48 (13.7%, 95% CI: 10.4-17.9) children were malaria microscopy-positive or PCR-positive and had an enlarged spleen at the same time. Sensitivity, specificity, positive (PPV) and negative predictive values (NPV) of spleen enlargement for malaria in this population are shown in Table 4 using microscopy or PCR as reference method. The Venn diagram showing relationship between spleen rate, microscopy and PCR positive children aged two to nine years is shown in Figure 3.

**Table 4:** Spleen enlargement as predictor for malaria in children two to nine years old

**PCR/microscopy (%)**					
	**Sensitivity**	**Specificity**	**PPV**	**NPV**	**Malaria prevalence**
Survey I	39.7/41.8	92.6/89.6	73.8/58.5	74.4/81.4	34.6/26.0
Survey II	13.3/25.0	92.5/91.0	44.4/36.4	70.5/85.5	30.9/17.0

### Winter survey II

From the 436 individuals included in the winter survey (Table 5) only 35 individuals (8.0%, 95% CI: 5.7-11.1) were slide-positive for malaria, however 116 (26.6%, 95% CI: 22.6-31.1) tested positive by PCR. *Plasmodium falciparum* accounted for 58.6%, *P. vivax* for 21.6%, *P. malariae* for 4.3%, and *P. ovale* for 0.9% of all PCR malaria-positive individuals, the remaining 14.7% were mixed infections.

**Table 5:** Malaria prevalence in three Bandarban subdistricts (Survey II)

**Bandarban district**		**PCR/Microscopy**					
	**No**	***P. f.***	***P. v.***	***P. m.***	***P. o.***	**Mixed**	**Negative**
Sex							
Male	178	38/17 (21.3/9.6%)	9/3 (5.1/1.7%)	1/0 (0.6/0.0%)	1/0 (0.6/0.0%)	8/0 (4.5/0.0%)	121/158 (68.0/88.8%)
Female	258	30/8 (11.6/3.1%)	16/5 (6.2/1.9%)	4/0 (1.6/0.0%)	0/0 (0.0/0.0%)	9/1 (3.5/0.4%)	199/244 (77.1/94.6%)
**Male/Age** (years)							
0-7	42	9/6 (21.4/14.3%)	4/2 (9.5/4.8%)	0/0 (0.0/0.0%)	0/0 (0.0/0.0%)	0/0 (0.0/0.0%)	29/34 (69.0/81.0%)
8-14	20	4/4 (20.0/20.0%)	1/0 (5.0/0.0%)	0/0 (0.0/0.0%)	0/0 (0.0/0.0%)	5/0 (25/0.0%)	10/16 (50.0/80.0%)
15-21	10	1/0 (10.0/0.0%)	1/0 (10.0/0.0%)	0/0 (0.0/0.0%)	0/0 (0.0/0.0%)	1/0 (10/0.0%)	7/10 (70.0/100%)
22-50	72	17/5 (23.6/6.9%)	2/1 (2.8/1.4%)	1/0 (1.4/0.0%)	0/0 (0.0/0.0%)	1/0 (1.4/0.0%)	51/66 (70.8/91.7%)
>50	34	7/2 (20.6/5.9%)	1/0 (2.9/0.0%)	0/0 (0.0/0.0%)	1/0 (2.9/0.0%)	1/0 (2.9/0.0%)	24/32 (70.6/94.1%)
**Female/Age** (years)							
0-7	45	5/1 (11.1/2.2%)	1/2 (2.2/4.4%)	0/0 (0.0/0.0%)	0/0 (0.0/0.0%)	3/1 (6.7/2.2%)	36/41 (80/91.1%)
8-14	19	2/1 (10.5/5.3%)	3/0 (15.8/0.0%)	1/0 (5.3/0.0%)	0/0 (0.0/0.0%)	2/0 (10.5/0.0%)	11/18 (57.9/94.7%)
15-21	28	3/2 (10.7/7.1%)	2/0 (7.1/0.0%)	0/0 (0.0/0.0%)	0/0 (0.0/0.0%)	3/0 (10.7/0.0%)	20/26 (71.4/92.9%)
22-50	127	18/4 (14.2/3.1%)	9/3 (7.1/2.4%)	3/0 (2.4/0.0%)	0/0 (0.0/0.0%)	1/0 (0.8/0.0%)	96/120 (75.6/94.5%)
>50	39	2/0 (5.1/0.0%)	1/0 (2.6/0.0%)	0/0 (0.0/0.0%)	0/0 (0.0/0.0%)	0/0 (0.0/0.0%)	36/39 (92.3/100%)
**Subdistrict**							
Bandarban Sadar	273	49/21 (17.9/7.7%)	18/7 (6.6/2.6%)	4/0 (1.5/0.0%)	1/0 (0.4/0.0%)	9/0 (3.3/0.0%)	192/245 (70.3/89.7%)
Rowangchhari	96	15/4 (15.6/4.2%)	4/0 (4.2/0.0%)	1/0 (1.0/0.0%)	0/0 (0.0/0.0%)	6/1 (6.3/1.0%)	70/91 (72.9/94.8%)
Ruma	67	4/0 (6.0/0.0%)	3/1 (4.5/1.5%)	0/0 (0.0/0.0%)	0/0 (0.0/0.0%)	2/0 (3.0/0.0%)	58/66 (86.6/98.5%)

*Plasmodium falciparum* gametocytes were detected by microscopy in four (3.5%, 95% CI: 1.1-9.1) individuals of whom only one also had asexual parasites. Malaria prevalence within all participants in the winter survey (dry season) was not significantly (p = 0.117) lower compared to the summer survey.

No patient testing positive for malaria was febrile at the time of the survey, therefore 103 (88.8%, 95% CI: 81.3-93.7) were classified as asymptomatic and 13 (11.2%, 95% CI: 6.3-18.7) as oligosymptomatic (Table 3).

The geometric mean parasite densities were 487 asexual parasites/μl (95% CI: 281–845) for *P. falciparum* and 751 (95% CI: 298–1,894) for *P. vivax,* respectively. No statistically difference (p > 0.05) was found between the parasite rate in summer and winter survey.

From a total of 172 households, in 77 (44.8%, 95% CI: 37.3-52.5) at least one member tested positive for malaria, in 27 (15.7%, 95% CI: 1.4-7.8) households two individuals and in seven (4.1%, 95% CI: 1.8-8.5) households three or more people tested positive for malaria.

### Subdistricts/*Upazilas*

In the winter survey the highest malaria prevalence by subdistrict was found in Bandarban Sadar (29.7%, 95% CI: 24.4-35.5) followed by Rowangchhari (27.1%, 95% CI: 18.8-37.3), and the lowest malaria prevalence was detected in Ruma (13.4%, 95% CI: 6.7-24.5).

### Splenomegaly

Out of 97 children aged two to nine years, nine (9.3%, 95% CI: 4.6-17.3) had a palpable spleen and with 30.9% (N = 30; 95% CI: 22.2-41.2) the parasite prevalence by PCR in survey II was again high but not significantly different from survey I (p = 0.584). Malaria-positive PCR and an enlarged spleen at the same time were found in four (4.1%, 95% CI: 1.3-10.8) children two to nine years old. With 9.3% the spleen rate in winter was also lower than in summer (18.6%; p = 0.043). Sensitivity, specificity, PPV and NPV of spleen enlargement for malaria in the winter survey are shown in Table 4.

## Discussion

Malaria is a major public health problem in southeastern Bangladesh and remains one of the most common reasons for hospital admissions during the malaria season [15]. Mapping of high-risk areas is essential for planning health interventions [37]. The primary objective of this investigation was to establish detailed baseline data on the prevalence and distribution of malaria in both symptomatic and asymptomatic carriers of *Plasmodium* parasites in southeastern Bangladesh.

Three major observations can be deducted from these cross-sectional surveys. First, this study demonstrates that malaria is meso-endemic in the region and is concentrated in rural communities [38] where the intensity of transmission is largely dependent on environmental variables. Haque *et al*. have previously reported for this region that environmental factors, such as proximity to forest, household density, and elevation tend to be significantly and positively correlated with malaria risk [39]. The development of immunity is insufficient to prevent the effect of malaria on all age groups [35]. A spleen rate of 18.6% (proportions of splenomegaly >10% are considered to be related to malaria [35]) and malaria prevalence by microscopy of 14.2% in the rainy and 7.8% in dry season, show that malaria is meso-endemic throughout the year in this part of Bangladesh. The spleen rate and the higher malaria prevalence in children are further indicators of meso-endemicity and suggest that transmission occurs within the villages [35,38].

Second, malaria prevalence was significantly higher in the northern and eastern subdistricts that are located in the hilly areas bordering Myanmar and Rangamati, another endemic district, whereas endemicity was lower in western and southern subdistricts in or close to the coastal plains, which are more accessible and where access to health care facilities is better. The collected data indicate a level of malaria transmission similar to some African countries, such as Gabon [21], Somalia [40] or Mozambique [41]. Previous studies from India and Indonesia also detected similar parasite prevalence rates [42,43].

Third, a large reservoir of asymptomatic, parasitaemic individuals that are likely to act as a source of infection, was observed in both surveys. The absence of malaria-like symptoms may be an indication of a certain level of immunity in rural communities. Although generally considered to be a typical feature of malaria in highly endemic regions in Africa, a number of studies from Brazil, Peru, Thailand, Cambodia, Myanmar, Vietnam, eastern Indonesia, and Papua New Guinea have reported the existence of asymptomatic malaria infections outside of Africa and in areas with lower endemicity [34,43-49]. This survey indicates a similar proportion of asymptomatic malaria carriers as previously reported from Vietnam [50], Indonesia [43] and Cambodia [45].

Naturally acquired immunity against *P. falciparum*, which is believed to build up with long-term exposure to malaria and presents with lower parasite densities and fewer clinical malaria episodes in older children and adults, has been reported from endemic areas in Myanmar, eastern Indonesia and India [42]. In areas with lower transmission intensity, the age at which clinical immunity develops tends to shift to an older age [42]. Malaria prevalence, parasite density and the number of clinical malaria cases were higher in children under 15 years.

As previously reported by Alves *et al*. [46], asymptomatic carriers may act as a reservoir for parasites and are a likely source of infection. However, treatment is typically only provided to symptomatic patients as asymptomatic carriers are rarely seen and/or diagnosed at health care facilities. Particularly in times of malaria elimination, asymptomatic malaria carrier with a low parasitaemia will present new challenges for malaria control in the region where malaria diagnosis is mainly based on microscopy and RDT.

Limitations of this study include all potential shortcomings of a point-prevalence study as well as the potential bias arising from the fact that only one district has been surveyed which may not be representative for all 13 malaria endemic districts in Bangladesh. Secondly, there was some overlap with the previously reported cross-sectional survey based on RDTs [15], which, however, provides far less detailed epidemiological data.

## Conclusions

The surveys showed that there are areas in Bangladesh with prevalence rates comparable to those found in malaria-endemic regions of tropical Africa. This study indicates, in accordance with other studies from Southeast Asia, that there is still a substantial proportion of asymptomatic, parasitaemic individuals in Bangladesh that may act as a silent reservoir for malaria transmission. This has major implications for ongoing malaria control programs in Bangladesh that are based on prevention of infection through bed nets and treatment of symptomatic patients. Particularly considering ongoing elimination efforts these findings highlight the need for new intervention strategies targeting all infections, symptomatic as well as asymptomatic, for reducing potential sources of infection and for interrupting the transmission cycle. Based on this study, it is evident that malaria remains an important public health problem in the southeastern part of Bangladesh. Further research is needed to determine the role of asymptomatic individuals for malaria transmission in the area.

## Competing interests

The authors declare that they have no competing interests.

## Authors’ contributions

HN contributed to all steps from elaboration to the final review (study design, study coordination, overall supervision, data analysis and manuscript review). PS, KT, PSw, BL, MJ supervised and carried out the surveys (three study teams worked simultaneously during the summer survey) and data collection in the field. PS, BL and KT contributed to implementation, to data entry and data analysis. PS wrote the first draft of the manuscript. BL and KT contributed to the writing of the manuscript. RH helped to design the study protocol, monitored laboratory quality and corrected the manuscript. WAK participated in the coordination of patient samples collection in the field, drafted the manuscript and helped to analyze the data. WG helped to design the study protocol and revised the final manuscript. H-PF and VEH carried out the molecular genetic studies. All authors read, approved and gave their consent to the final manuscript.
